# Extrasynaptic CaMKIIα is involved in the antidepressant effects of ketamine by downregulating GluN2B receptors in an LPS-induced depression model

**DOI:** 10.1186/s12974-020-01843-z

**Published:** 2020-06-10

**Authors:** Xiao-Hui Tang, Guang-Fen Zhang, Ning Xu, Gui-Fang Duan, Min Jia, Ru Liu, Zhi-Qiang Zhou, Jian-Jun Yang

**Affiliations:** 1 Department of Anesthesiology, Zhongda Hospital, School of Medicine, Southeast University, Nanjing, Jiangsu China; 2 Minister of Education Key Laboratory of Model Animal for Disease Study, Model Animal Research Center, Nanjing University, Nanjing, Jiangsu China; 3 Department of Anesthesiology, Jinling Hospital, School of Medicine, Nanjing University, Nanjing, Jiangsu China; 4 Department of Anesthesiology, Pain and Perioperative Medicine, The First Affiliated Hospital of Zhengzhou University, Zhengzhou, Henan China

**Keywords:** Ketamine, Antidepressant, Extrasynaptic, CaMKIIα, GluN2B

## Abstract

**Background:**

A subanesthetic dose of ketamine provides rapid and effective antidepressant effects, but the molecular mechanism remains elusive. It has been reported that overactivation of extrasynaptic GluN2B receptors is associated with the antidepressant effects of ketamine and the interaction between GluN2B and calcium/calmodulin-dependent protein kinase IIα (CaMKIIα) is important for GluN2B localization and activity. Here, we tested whether changes of CaMKIIα and GluN2B are involved in the antidepressant effects of ketamine.

**Methods:**

Lipopolysaccharide (LPS) was injected intraperitoneally (i.p.) into male C57BL/6 mice. For the interventional study, mice were administrated with ketamine (10 mg/kg, i.p.) or a CaMKIIα inhibitor KN93. Behavioral alterations were evaluated by open-field, novelty-suppressed feeding, and forced-swimming tests. Physiological functions were evaluated by the body weight and fur coat state of mice. The levels of p-CaMKIIα, CaMKIIα, p-GluN2B, GluN2B, p-CREB, CREB, BDNF, GluR1, and GluR2 in the hippocampus were detected by western blotting. The interaction between GluN2B and CaMKIIα was studied using immunoprecipitation assay and small interfering RNA (siRNA) assays. The colocalizations of GluN2B/PSD95 and p-GluN2B/PSD95 were detected by immunofluorescence. The long-term potentiation (LTP) in SC-CA1 of the hippocampus was detected by electrophysiology.

**Results:**

LPS injection induced depression-like behaviors, which were accompanied by significant increases in extrasynaptic p-CaMKIIα expression, extrasynaptic GluN2B localization, and phosphorylation and decreases in p-CREB, BDNF, and GluR1 expressions and LTP impairment. These changes were prevented by ketamine administration. Immunoprecipitation assay revealed that LPS induced an increase in the p-CaMKIIα–GluN2B interaction, which was attenuated by ketamine administration. SiRNA assay revealed that CaMKIIα knockdown reduced the level and number of clusters of GluN2B in the cultured hippocampal neurons. KN93 administration also reduced extrasynaptic p-CaMKIIα expression, extrasynaptic GluN2B localization, and phosphorylation and exerted antidepressant effects.

**Conclusion:**

These results indicate that extrasynaptic CaMKIIα plays a key role in the cellular mechanism of ketamine’s antidepressant effect and it is related to the downregulation of extrasynaptic GluN2B localization and phosphorylation.

## Background

Depression is a common clinical psychiatric disease, which affects about 16% of the global population and places a serious social burden [1]. However, traditional antidepressants often require several weeks or months of continuous medication to achieve their full therapeutic effect [2]. Therefore, it is urgent to find novel antidepressant drugs that act rapidly and effectively. A single subanesthetic dose of ketamine is known to produce rapid and sustained effect [3]. However, the molecular mechanism underlying ketamine’s antidepressant effects remains to be elucidated.

Calcium/calmodulin-dependent protein kinase II (CaMKII) is a Ser/Thr protein kinase that is highly abundant in the brain. The CaMKII family of proteins comprises 28 similar isoforms that are derived from four genes (α, β, γ, and δ) [4]. CaMKIIα is the predominant isoform in the hippocampus and is central to the transmission of synaptic signals and the regulation of synaptic morphology [5]. Preclinical studies have shown that stress-induced activation of hypocretin/orexin receptors produces depression-like behaviors via an increase in p-CaMKIIα in mice and that siRNA-mediated inhibition of CaMKIIα counteracted the stress-induced depression-like behaviors [6]. Furthermore, fluoxetine, a typical antidepressant, reduces the binding of transcription factor ΔfosB to CaMKIIα promoter and reduces CaMKII expression, and CaMKII overexpression blocks the antidepressant effects of fluoxetine in a chronic social defeat stress model [7]. In a recent study, it has been demonstrated that ketamine administration induces CaMKII autoinhibition (pT305 phosphorylation) first and then autoactivation (pT286), whereas CaMKII inhibitor tatCN21 pre-treatment prevented ketamine’s antidepressant effects [8]. Together, these studies suggest that CaMKIIα plays a critical role in the stress-induced depression-like behaviors and the antidepressant effects of ketamine. However, whether CaMKIIα is involved in ketamine’s antidepressant effect in the lipopolysaccharide (LPS)-induced depression model is unclear.

*N*-methyl-d-aspartate receptors (NMDARs) are ionotropic glutamate receptors consisting of an essential GluN1 subunit and one or more regulatory GluN2 subunits (GluN2A–D). Because of the different localization of NMDARs on the synapse, they can be further divided into synaptic NMDARs and extrasynaptic NMDARs. Immunofluorescence and immunogold studies identify that GluN2B subunit is more abundant in extrasynaptic fractions [9, 10]. Furthermore, knocking out the GluN2B subunit in principal cortical neurons of mouse occludes ketamine’s antidepressant actions [11]. Meanwhile, GluN2B-selective antagonists produce antidepressant effects in rodent models of depression [12] and improve clinical symptoms of patients with depression [13]. The above results suggest that GluN2B receptors are essential for ketamine’s antidepressant effects, yet the upstream and downstream signaling involved in GluN2B remains unclear.

CaMKIIα is activated when calcium ions enter the cell via various ion channels on the membrane and the activation of CaMKIIα ultimately promotes autophosphorylation at threonine 286 (p-CaMKIIα), which binds to GluN2B and phosphorylates GluN2B at the S1303 site (p-GluN2B) [14, 15]. The synaptic binding of CaMKIIα/GluN2B has been intensively studied [16], and the interaction between GluN2B and CaMKIIα is important for synaptic CaMKIIα localization and activity [14, 17]. However, the role of extrasynaptic CaMKIIα and GluN2B in the antidepressant effects of ketamine has not been intensively investigated. Hence, in this study, we investigated the role of changes in extrasynaptic CaMKIIα and GluN2B, as well as the interaction between CaMKIIα and GluN2B, in the antidepressant effects of ketamine using an LPS-induced depression model.

## Materials and methods

### Animals

Male adult C57BL/6J mice (25–30 g) were purchased from the Model Animal Research Center (MARC) of Nanjing University, Nanjing, China. Four to five individuals were placed in each cage, and the mice were housed in a room temperature of 23 ± 1 °C, with a 12-h light/dark cycle. The mice were free to food and water. All animal experiments and related operations were carried out in accordance with the Guideline for the Care and Use of Laboratory Animals from the National Institutes of Health, USA.

### LPS-induced depression model

In this experiment, the dose of LPS (1 mg/kg) was intraperitoneally (i.p.) injected based on one previous study [18]. LPS was dissolved in physiological saline and then injected at a volume of 10 ml/kg in all conditions. The solution is ready for use and injection of LPS was scheduled at a fixed time (09:00 and 10:00 a.m.).

### Experimental design and drug administration

#### Experiment 1

**Fig. 1:**
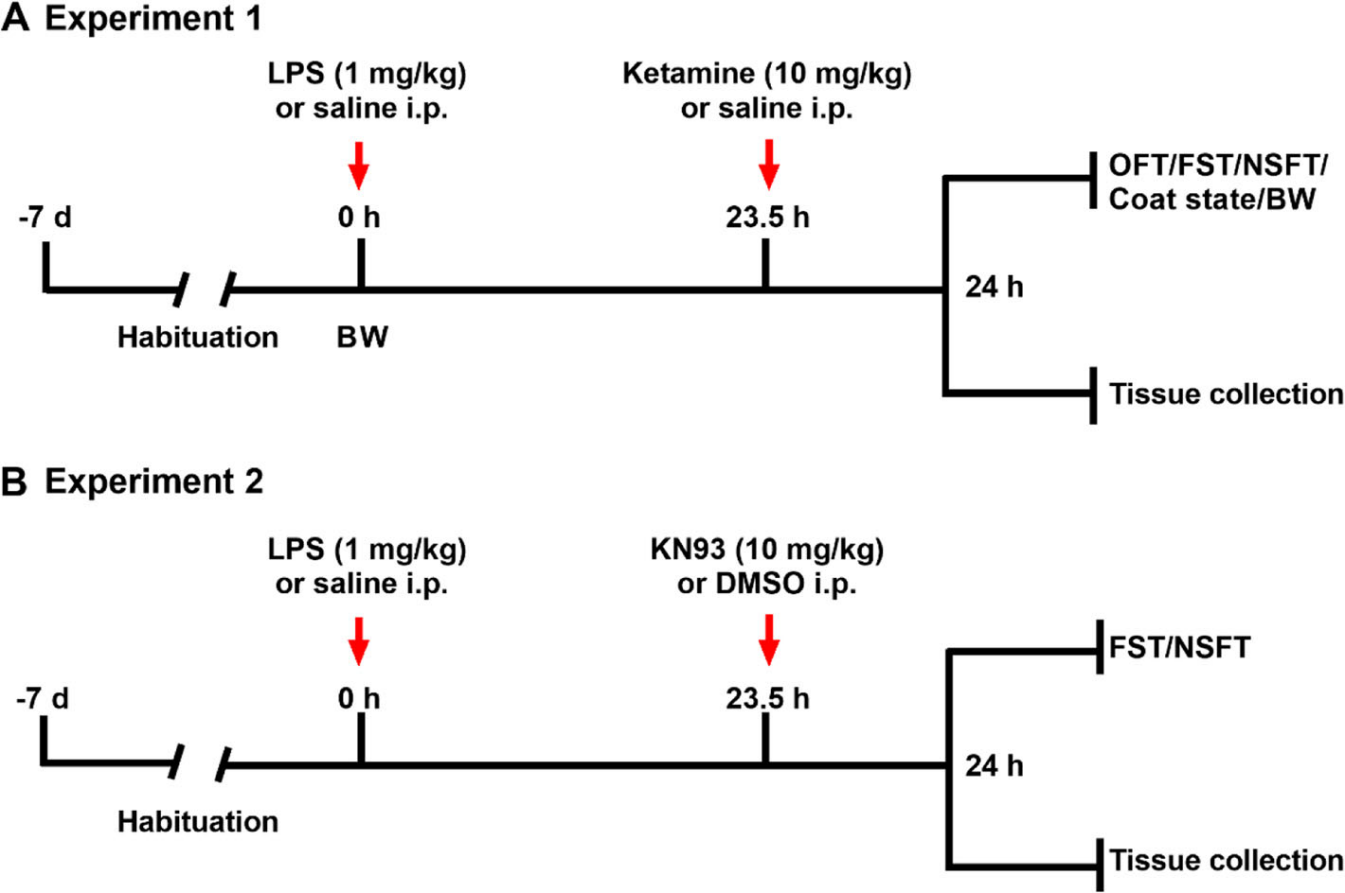
Timeline of drug injection, behavioral test, and tissue collection: experiment 1 (**a**) and experiment 2 (**b**). BW, body weight; DMSO, dimethyl sulfoxide; FST, forced-swimming test; KN93, C_26_H_29_ClN_2_O_4_S.H_3_O_4_P; LPS, lipopolysaccharide; NSFT, novelty-suppressed feeding test; OFT, open-field test

Mice were randomly numbered and then divided into 4 groups: the Sal + Sal group, the LPS + Sal group, the Sal + Ket group, or the LPS + Ket group. First, LPS (1 mg/kg) or an equal volume of physiological saline was injected i.p. between 09:00 and 10:00 a.m. After 23.5 h, ketamine (10 mg/kg) or an equal volume of physiological saline was injected i.p. The open-field test (OFT), novelty-suppressed feeding test (NSFT), forced swim test (FST), body weight, and fur coat state of mice were evaluated 0.5 h after ketamine administration. In each group of mice, half of the animals underwent behavioral tests and the other half subjected to biochemical tests. The flow chart of experiment 1 is shown in Fig. 1a.

#### Experiment 2

Mice were randomly numbered and then divided into 4 groups: the Sal + dimethyl sulfoxide (DMSO) group, the LPS +DMSO group, the Sal + KN93 group, or the LPS + KN93 group. First, LPS (1 mg/kg) or an equal volume of physiological saline was injected i.p. between 09:00 and 10:00 a.m. After 23.5 h, KN93 (10 mg/kg) or an equal volume of DMSO was injected i.p. The selected dose of KN93 in this experiment is based on the pre-experiment results. The NSFT and FST were performed 0.5 h after KN93 administration. In each group of mice, half of the animals underwent behavioral tests and the other half subjected to biochemical tests. The flow chart of experiment 2 is shown in Fig. 1b.

Both ketamine hydrochloride (Gutian Pharmaceutical Company, China) and LPS (L-2880, Sigma, USA) were dissolved in physiological saline. KN93 (S7423, Selleckchem, USA) was dissolved in 1% DMSO.

### Behavioral tests

Behavioral experiments are carried out in quiet rooms using an XR-XZ301 instrument (Xinruan Corporation, Shanghai, China). All behavioral data were analyzed by a researcher blinded to the experimental grouping.

### Open-field test

Mice were placed in a white experimental box (40 cm × 40 cm × 40 cm; length × width × height) and allowed to move freely for 5 min. The entire process was automatically tracked by a camera placed above the experimental box. The total distance traveled was considered to be a measure of the ability of the mouse to move. The time spent in the center and the number of entries into the center were considered to be a measure of the level of anxiety in mice. At the end of each test, wipe the instrument with 75% alcohol to avoid the effect of the residual odor of the previous animal on the test results.

### Novelty-suppressed feeding test

The mice were fasted 12 h before the experiment and the drinking water was not limited [19]. Before the experiment, two pieces of chow were placed in the center of a plastic box (40 cm × 40 cm × 40 cm; length × width × height). Then, mice were placed in the test box and allowed to move freely for 10 min. The time required for the mouse to enter the box to eat food for the first time is the feeding latency. The feeding latency was considered to be a measure of the level of depression in mice. At the end of the experiment, mice were placed in a cage with pre-weighed food, and the consumption of this food was recorded for 15 min.

### Forced-swimming test

Mice were placed in a glass container (18-cm diameter × 28-cm height) of water (15 cm; 25 ± 1 °C) and allowed to swim under normal light for 6 min and recorded the total immobility time of the last 4 min [20]. The definition of immobility time refers to the time when the mouse passively floated with no additional activity or with no other movements except those to maintain balance in the water. After the experiment, the mouse body was wiped dry with absorbent paper and placed back in the original cage. Replace the water at the end of each test.

### Physiological changes

The body weight change was monitored. For fur coat state, seven different body parts were evaluated: head, neck, dorsal and ventral coat, tail, forepaws, and hindpaws. For each area, a health state was noted 3 and damaged state with piloerection and/or dirty fur was noted 1. Intermediate state was noted 2. The total score of the coat state is the average of scores from the above seven body parts [21].

### Western blot analysis

#### Tissue preparation and subcellular fractionation

The levels of p-CaMKIIα, CaMKIIα, p-GluN2B, and GluN2B in the synaptic and extrasynaptic fractions of the hippocampus were assessed by western blotting. The separation methods of the synaptic and extrasynaptic fractions refer to previous studies [22]. Briefly, the hippocampus of adult mice was dissected and homogenized in an ice-cold sucrose buffer (in mM): 320 sucrose, 10 Tris (pH 7.4), 1 EDTA, 1 Na_3_VO_4_, 5 NaF, 1 EGTA, and 1× protease inhibitor cocktail, and then centrifuged at 1000×*g* for 10 min at 4 °C. The supernatant (S1) was collected and centrifuged at 10,000×*g* for 20 min at 4 °C. Then, the pellet (P2) was saved and resuspended in sucrose buffer. Then, the pellet (P2) was centrifuged at 10,000×*g* for 20 min at 4 °C and repeated twice. Then, the pellet (P2) was resuspended in an ice-cold Triton X-100 buffer (in mM): 10 Tris (pH 7.4), 1 EDTA, 1 Na_3_VO_4_, 5 NaF, 1 EGTA, and 0.5 % Triton X-100. This pellet (P2) was rotated slowly 20 min at 4 °C. Then, the pellet (P2) was centrifuged at 32,000×*g* for 20 min at 4 °C. The supernatant and pellet comprised the extrasynaptic fraction (non-PSD) and synaptic fraction (PSD), respectively. The separation of the synaptic and extrasynaptic fraction was demonstrated by the distribution of postsynaptic density-95 (PSD95) and synaptophysin. The protein concentration of the four groups was measured by bicinchoninic acid (BCA) protein assay and then adjusted to the same level.

#### Total protein preparation

The levels of p-CREB, CREB, and BDNF in the total protein of the hippocampus were assessed by western blotting. Briefly, the hippocampus of adult mice was harvested on the ice and then homogenized in an ice-cold protein lysis buffer (in mM): 150 NaCl, 50 Tris-HCl, 0.1% SDS, 1% Triton X-100, 1% sodium deoxycholate, 1 NaF, 1 Na_3_VO_4_, 1 PMSF, and 1× protease inhibitor cocktail. The homogenate was centrifuged at 10,000 rpm for 10 min at 4 °C. Then, the protein concentration of the four groups was measured by BCA protein assay and then adjusted to the same level.

#### Synaptosome preparation

The levels of GluR1 and GluR2 in the synaptosome of the hippocampus were assessed by western blotting. The separation methods of the synaptosome fractions refer to previous studies [23]. Briefly, the hippocampus of adult mice was harvested on the ice and then homogenized in an ice-cold sucrose solution (in mM): 320 sucrose, 20 HEPES (pH 7.4), 1 Na_3_VO_4_, 1 EDTA, 5 NaF, and 1× protease inhibitor cocktail, and then centrifuged at 2800 rpm for 10 min at 4 °C. The supernatant was collected and centrifuged at 12,000 rpm for 10 min at 4 °C. Then, the pellet (synaptosome fraction) was saved and resuspended in an ice-cold protein lysis buffer (in mM): 150 NaCl, 50 Tris-HCl (pH 7.4), 1% Triton X-100, 0.1% SDS, 1 Na_3_VO_4_, 2 EDTA, 5 NaF, and 1× protease inhibitor cocktail. The protein concentration of the four groups was measured by BCA protein assay and then adjusted to the same level.

Protein (10-20 μg/well) were separated by 8–12% SDS-PAGE gels and then transferred to polyvinylidene fluoride membranes. Five percent non-fat milk was used to incubate the membranes for 2 h at room temperature. After this, the membranes were incubated primary antibody overnight at 4 °C. The selected primary antibody is as follows: anti-BDNF (Abcam, Cambridge, UK), anti-CaMKIIα (phospho-T286) (Abcam, Cambridge, UK), anti-CaMKIIα (Abcam, Cambridge, UK), anti-CaMKIIβ (Abcam, Cambridge, UK), anti-CREB (Cell Signaling, MA, USA), anti-GAPDH (ProteinTech, USA), anti-β-actin (GeneTex, USA), anti-PSD95 (Millipore, MA, USA), anti-synaptophysin (Millipore, MA, USA), anti-NMDAR2B (phospho-S1303) (Abcam, Cambridge, UK), anti-Glutamate Receptor 1 (Abcam, Cambridge, UK), anti-NMDAR2B (Abcam, Cambridge, UK), anti-p-CREB (Cell Signaling, MA, USA), and anti-Glutamate Receptor 2 (Abcam, Cambridge, UK). The membranes were washed three times in TBST and then incubated in the corresponding secondary antibody (goat anti-rabbit or mouse and rabbit anti-goat; Bioworld Technology, MN, USA) for 2 h at room temperature. Finally, the protein bands were visualized by chemiluminescence method. The mean gray value of each protein band was quantified by Image J (NIH, Bethesda, MD, USA).

### Immunofluorescence

Mice were anesthetized with 1% sodium pentobarbital (50 mg/kg, i.p.; Sigma, USA), followed by systemic perfusion of the mice via the left ventricle using physiological saline and 4% PFA. Then, the whole brain of the mice was taken out and dipped in 4% PFA for 4–6 h at 4 °C. After that, the brain was dehydrated in 20% and 30% sucrose at 4 °C, respectively. OCT was used to embed the brain when it sinks to the bottom. A 10-μm-thick coronal sections of the hippocampus were cut and immediately adhered to the slide. The slides were immersed in the PBS for 10 min to wash away the OCT during embedding. Next, the slides were blocked with 10% goat serum for 2 h at room temperature. After that, slides were incubated in primary antibodies diluted with 5% BSA overnight at 4 °C: anti-NMDAR2B (phospho-S1303) (Abcam, Cambridge, UK), anti-NMDAR2B (Abcam, Cambridge, UK), and anti-PSD95 (Millipore, MA, USA). The slides were washed with PBS (with 5 ‰ Triton X-100) for 3 × 5 min. Then, the slides were incubated in corresponding secondary antibodies (Alexa Fluor 488/549 goat anti-rabbit or mouse; Bioworld Technology, MN, USA) for 2 h at room temperature. After washing with PBS (with 5 ‰ Triton X-100) for 3 × 5 min, the slides were incubated with DAPI to label nuclei. A confocal scanning microscope (Carl Zeiss, LSM880, Germany) was used to obtain the fluorescence images of the region of interest. The Pearson’s coefficient colocalization was used to quantify the colocalization of two confocal immunofluorescences, which was calculated by a plug-in package in Image J (NIH, Bethesda, MD, USA).

### Immunoprecipitation

Immunoprecipitation was used to detect the interaction between p-CaMKIIα and GluN2B. Immunoprecipitation from the extrasynaptic fractions of the hippocampus was performed with rabbit NMDAR2B (2–3 μg) antibody [24]. Briefly, the extrasynaptic fractions of the hippocampus were obtained as described above. The extracts were precleared by adding nonspecific control immunoglobulin G (1 μg) and 20 μl of Protein G Sepharose (Sigma, USA). The supernatant was collected and incubated with nonspecific IgG (2 μg) or rabbit anti-NMDAR2B (2 μg; Abcam, Cambridge, UK) overnight at 4 °C. The next day, they were mixed with the addition of 40 μl of Protein G Sepharose (Sigma, USA) and then rotated slowly for 4 h at 4 °C. After that, the beads were washed three times in buffer A (in mM): 150 NaCl, 50 Tris-HCl, 0.1% Triton X-100, and 1 EDTA. Then, the beads were washed three times in buffer B (in mM): 300 NaCl, 50 Tris-HCl, 0.1% Triton X-100, and 1 EDTA. Finally, the beads were denatured in SDS buffer and separated by SDS-PAGE.

### Primary hippocampal neuron cultures

Primary hippocampal neurons were prepared and processed as described previously with minor modifications [25]. The hippocampus from postnatal (P0-P1) C57BL/6 mice were rapidly and aseptically dissected in ice-cold DMEM medium, followed by removal of meninges and mincing into small pieces. The hippocampal tissue was then digested in 0.25% trypsin for 15 min at 37 °C in a humidified atmosphere of 5% CO_2_ and 95% air. The hippocampal tissue was washed twice with DMEM medium (including FBS, F12); then, the tissue was gently blown (not more than 30 times) and then allowed to stand for 10 min. The supernatant was gently resuspended in culture medium and plated at 40,000–50,000 cells per cm^2^ on Poly-l-lysine-coated (Sigma-Aldrich). Every 3 days, half of the medium was replaced with freshly prepared medium.

### Primary neuron transfection and siRNA

Hippocampal neurons were transfected at DIV10 using Lipofectamine 2000 (Invitrogen, Carlsbad, CA) according to the manufacturer’s recommendations. For CaMKIIα knockdown, cells were transfected with 50 nM CaMKIIα siRNA for 24 h. Control siRNA and CaMKIIα siRNA (R10043.8) were from Guangzhou RiboBio Co., Ltd. The culture treatments were conducted 24 h after transfection.

### Immunoblotting and immunocytochemistry

For cell western blotting assays, hippocampal neurons were washed gently with PBS for three times and then harvested in an ice-cold protein lysis buffer (in mM): 150 NaCl, 50 Tris-HCl, 0.1% SDS, 1% Triton X-100, 1% sodium deoxycholate, 1 NaF, 1 Na_3_VO_4_, 1 PMSF, and 1× protease inhibitor cocktail. The homogenate was centrifuged at 10,000 rpm for 10 min at 4 °C. Then, the protein concentration was measured by BCA protein assay and then adjusted to the same level. The rest of the operations are the same as tissue western blotting assays.

Hippocampal neurons were fixed with a 4% PFA/4% sucrose mixture in PBS that was preheated to 37 °C in advance for 10 min at room temperature. The neurons were then permeabilized with 0.1% (vol/vol) Triton X-100 in PBS for 10 min at room temperature and washed gently with PBS for three times. The neurons were blocked with ready-to-use normal goat serum for 1 h at room temperature and subsequently incubated at 4 °C overnight with primary antibodies. The primary antibodies used were anti-CaMKIIα (Abcam, Cambridge, UK) and anti-NMDAR2B (Abcam, Cambridge, UK).

After washing with PBS for 3 × 5 min, the neurons were exposed to the secondary antibodies Alexa fluor 488/549 goat anti-rabbit (1:100; Bioworld Technology, St. Louis Park, MN, USA), and DAPI to label nuclei (1:1000; Sigma, St. Louis, MO, USA) for 2 h at room temperature. Fluorescence images were obtained by confocal scanning microscopy (Carl Zeiss, LSM880, Germany). Numbers of GluN2B puncta as well as the mean intensity of fluorescent signals (CaMKIIα and GluN2B) in individual neurons were measured using the Image J function “Analyze > measure.”

### Electrophysiological recording

The mice were anesthetized and the whole brain was taken out on the ice. The brain was quickly dipped in a pre-oxygenated (95% O_2_/5% CO_2_) cutting solution (in mM): 2.6 KCl, 1.25 NaH_2_PO_4_, 26 NaHCO_3_, 0.5 CaCl_2_, 5 MgCl_2_, 212 sucrose and 10 dextrose for 2 min. In the cutting solution, a 300-μm-thick coronal slices of the hippocampus were cut out. Then, the slices were immediately transferred to the artificial cerebrospinal fluid (ACSF) (in mM): 124 NaCl, 5 KCl, 1.25 NaH_2_PO_4_, 26 NaHCO_3_, 2 CaCl_2_, 2 MgCl_2_, and 10 dextrose for incubation. The hippocampal slices were then kept at 26 °C for 1 h before recording. In order to induce LTP, concentric bipolar tungsten electrodes and the recording pipettes were placed in the Schaffer collateral–commissural fibers and the stratum radiatum of hippocampal CA1 region, respectively. During the recording period, ACSF was perfused continuously and the picrotoxin (0.1 mM) and APV (80 μM) were added to the ASCF to block GABA_A_ and NMDA receptors, respectively. In this study, LTP was evoked by high-frequency stimulation (HFS; three trains of 100 Hz with a 10-s interval between each train). A steady baseline was recorded for at least 10 min before the induction of LTP. The field excitatory postsynaptic potential (fEPSP) slope between 10% and 90% was used to indicate the fEPSP magnitude. Data are normalized to mean baseline value and shown as mean ± S.E.M. Slices were considered to demonstrate LTP if the amplitude of the fEPSP was increased by at least 15% compared to baseline. The signal was amplified with pClamp 700B amplifier (Axon Instruments, Foster City, CA), acquired at 10 kHz and filtered at 2 kHz.

### Statistical analysis

Data are shown as mean ± SEM. SPSS software (version 25.0, IL, USA) and GraphPad Prism (version 7, CA, USA) were used for statistical analyses. For comparisons of two groups, the two-tailed unpaired *t* test was used for statistical analysis. For three or more groups of samples, Shapiro–Wilk test and Levene’s test were used for the distribution and variance analysis of the data. When data were not normally distributed, the differences among groups were compared by Kruskal–Wallis one-way ANOVA followed by Bonferroni’s correction. When data were normally distributed, the differences among groups were compared by two-way ANOVA followed by Bonferroni’s multiple comparisons test. LPS injection and ketamine or KN93 administration were considered two independent factors. *P* < 0.05 indicates that the difference is statistically significant.

## Results

### Ketamine improved LPS-induced depression-like behaviors

**Fig. 2:**
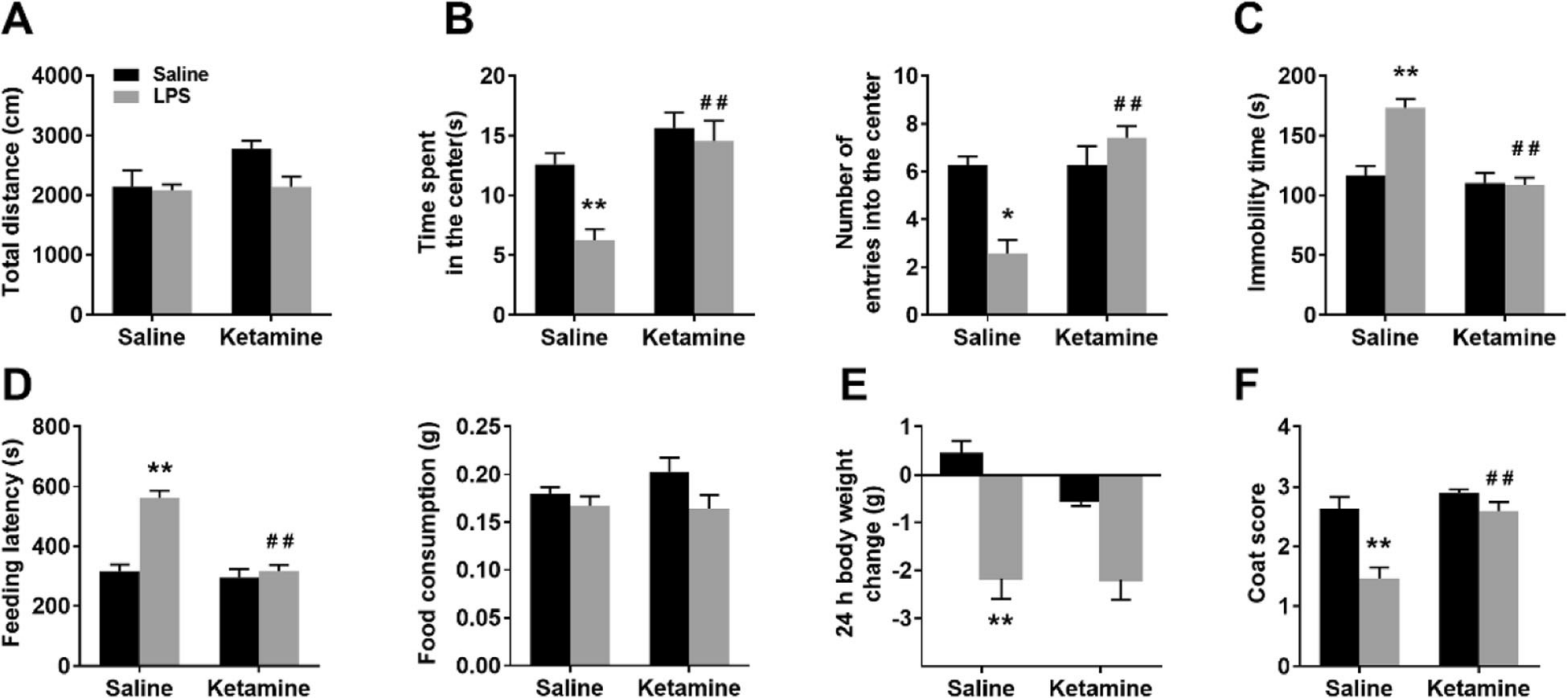
Ketamine administration rescued LPS-induced depression-like behaviors. **a** In OFT, there was no significant difference in total distance within 5 min among the four groups. **b** In the OFT, ketamine administration prevented LPS-induced decrease in time spent in the center and the number of entries into the center. **c** In the NSFT, ketamine administration prevented LPS-induced increase in feeding latency, and the total food consumption was not different among the four groups. **d** In the FST, ketamine administration prevented LPS-induced increase in immobility time. **e** Ketamine administration did not affect LPS-induced decrease in body weight change in the mice. **f** Ketamine administration improved the poor coat state of mice induced by LPS. Data are presented as mean ± SEM (*n* = 7–10 mice per group). *p < 0.05, **p < 0.01 vs the Sal + Sal group; ^##^*p* < 0.01 vs the LPS + Sal group. Sal, saline

In the OFT, no significant difference in the total traveled distance within 5 min was observed in the four groups, indicating that LPS injection and ketamine administration did not affect the locomotor activity of mice (Fig. 2a; interaction: LPS × ketamine, *F*_1,24_ = 2.54, *P* > 0.05; LPS: *F*_1,24_ = 3.735, *P* > 0.05; ketamine: *F*_1,24_ = 3.663, *P* > 0.05; LPS is LPS + Sal vs Sal + Sal; ketamine is LPS + Ket vs LPS + Sal). LPS injection decreased the time spent in the center (interaction: LPS × ketamine, *F*_1,24_ = 4.40, *P* < 0.05; LPS: *F*_1,24_ = 8.869, *P* < 0.01; ketamine: *F*_1,24_ = 20.273, *P* < 0.01) and the number of entries into the center (interaction: LPS × ketamine; *F*_1,24_ = 18.253, *P* < 0.05; LPS: *F*_1,24_ = 5.116, *P* < 0.05; ketamine: *F*_1,24_ = 18.253, *P* < 0.01), both of these effects were rapidly reversed by ketamine administration (Fig. 2b). In the FST, LPS injection increased the immobility time, which was reversed by ketamine administration (Fig. 2c; interaction: LPS × ketamine, *F*_1,24_ = 15.13, *P* < 0.05; LPS: *F*_1,24_ = 13.414, *P* < 0.01; ketamine: *F*_1,24_ = 22.327, *P* < 0.01). In the NSFT, ketamine administration reversed LPS-induced increase in the feeding latency (interaction: LPS × ketamine, *F*_1,24_ = 21.226, *P* < 0.05; LPS: *F*_1,24_ = 30.552, *P* < 0.01; ketamine: *F*_1,24_ = 30.165, *P* < 0.01), and the total food consumption (interaction: LPS × ketamine, *F*_1,24_ = 1.126, *P* > 0.05; LPS: *F*_1,24_ = 4.504, *P* > 0.05; ketamine: *F*_1,24_ = 0.681, *P* > 0.05) in the four groups were not affected (Fig. 2d). Ketamine administration did not affect LPS-induced decrease in body weight change in the mice (Fig. 2e; interaction: LPS × ketamine, *F*_1,32_ = 0.871, *P* > 0.05; LPS: *F*_1,32_ = 0.959, *P* < 0.01; ketamine: *F*_1,32_ = 17.855, *P* > 0.05). LPS induced a poor coat state (indicated by a decreased score) in mice that was attenuated by ketamine administration (Fig. 2f; interaction: LPS × ketamine, *F*_1,24_ = 7.360, *P* > 0.05; LPS: *F*_1,24_ = 21.630, *P* < 0.01; ketamine: *F*_1,24_ = 19.293, *P* < 0.01). In summary, these behavioral results indicated that ketamine (10 mg/kg) administration can eliminate the depression and anxious-like behaviors caused by LPS injection without affecting the locomotor activity of mice.

### Ketamine reversed LPS-induced extrasynaptic CaMKIIα activity in the hippocampus

**Fig. 3:**
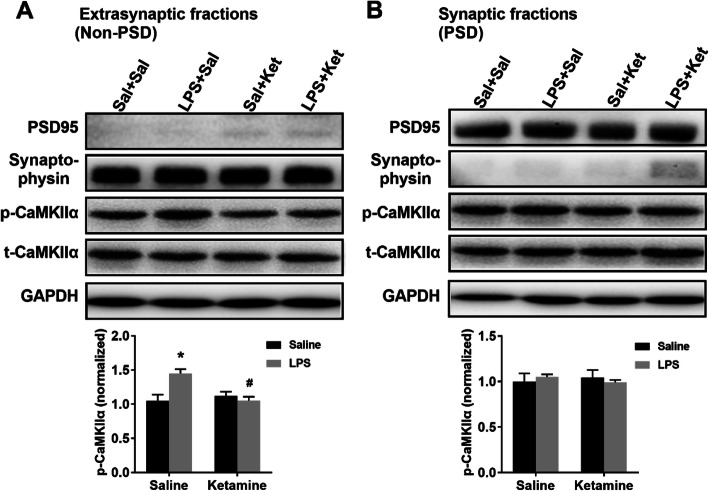
Ketamine reversed LPS-induced extrasynaptic CaMKIIα activity in the hippocampus. The levels of CaMKIIα and p-CaMKIIα in extrasynaptic and synaptic fractions of the hippocampus were determined by western blotting. **a** Ketamine administration reversed LPS-induced increase in extrasynaptic p-CaMKIIα level in the hippocampus. **b** There was no significant difference in synaptic p-CaMKIIα level in the hippocampus among the four groups. Data are presented as mean ± SEM (*n* = 4–6 mice per group). *p < 0.05 vs the Sal + Sal group; ^#^*p* < 0.05 vs the LPS + Sal group

In the extrasynaptic fractions of the hippocampus, ketamine administration reversed the elevated level of p-CaMKIIα induced by LPS injection (Fig. 3a; interaction: LPS × ketamine, *F*_1,12_ = 11.495, *P* < 0.05; LPS: *F*_1,12_ = 5.710, *P* < 0.05; ketamine: *F*_1,12_ = 5.604, *P* < 0.05). In the synaptic fractions of the hippocampus, no significant difference in the level of p-CaMKIIα was observed in the four groups (Fig. 3b; interaction: LPS × ketamine, *F*_1,12_ = 0.426, *P* > 0.05; LPS: *F*_1,12_ = 0.000, *P* > 0.05; ketamine: *F*_1,12_ = 0.015, *P* > 0.05).

### Ketamine reversed LPS-mediated extrasynaptic GluN2B localization and phosphorylation in the hippocampus

**Fig. 4:**
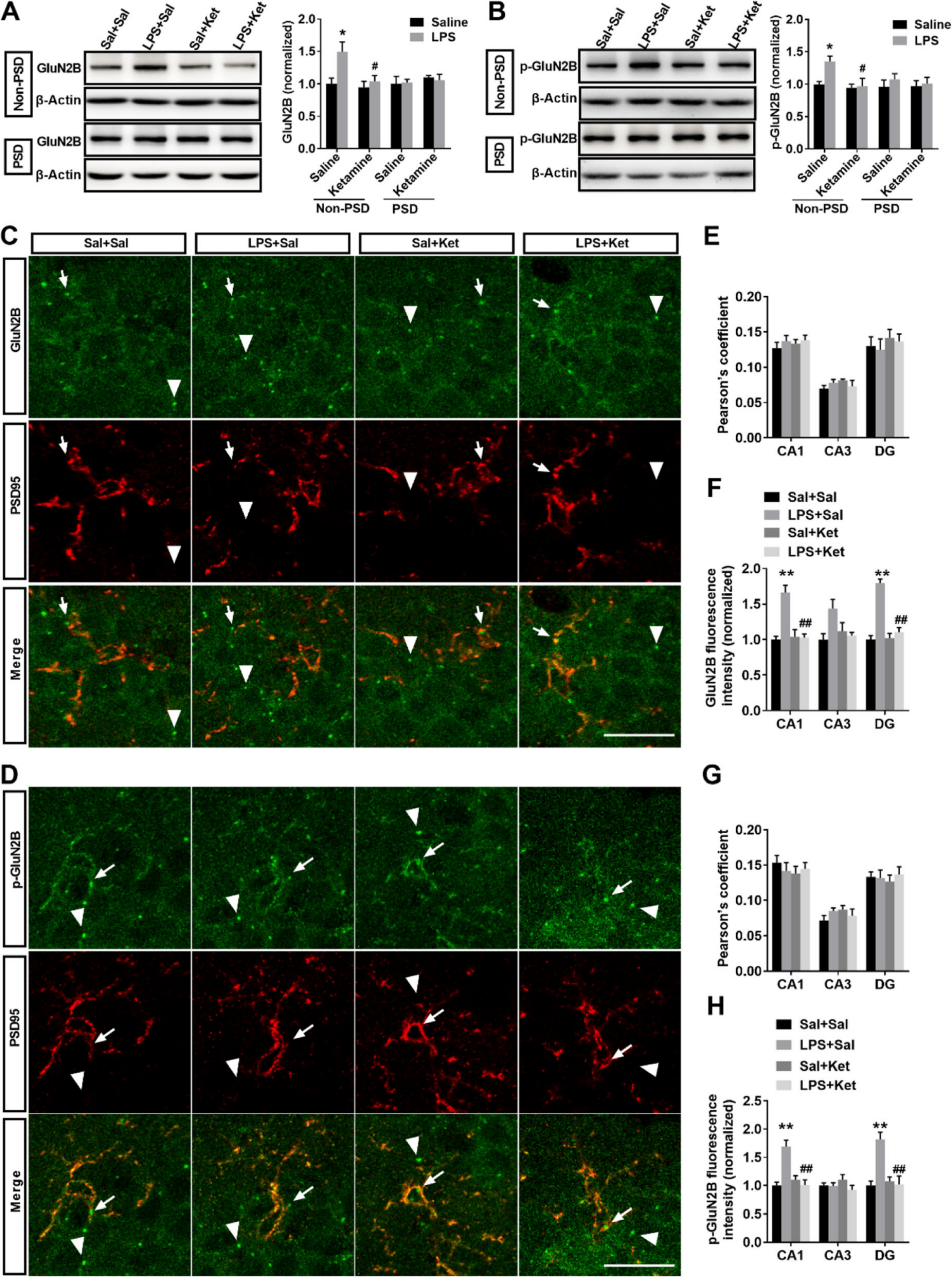
Ketamine reversed LPS-induced increase in extrasynaptic GluN2B localization and phosphorylation in the hippocampus. The levels of GluN2B and p-GluN2B in extrasynaptic and synaptic fractions of the hippocampus were determined by western blotting and immunofluorescence. **a** Ketamine administration reversed LPS-induced increase in extrasynaptic GluN2B level but did not affect synaptic GluN2B level in the hippocampus. **b** Ketamine administration reversed LPS-induced increase in extrasynaptic p-GluN2B level but did not affect synaptic p-GluN2B level in the hippocampus. **c** Immunofluorescent images show the colocalization of PSD95 (red) and surface GluN2B receptor (green) in CA1 regions of the hippocampus. Arrow indicates colocalization, and triangle indicates no colocalization. **d** Immunofluorescent images show the colocalization of PSD95 (red) and p-GluN2B (green) in CA1 regions of the hippocampus. Arrow indicates colocalization, and triangle indicates no colocalization. **e** Pearson’s correlation coefficient of PSD95 and GluN2B was not significantly different among the four groups in CA1, CA3, and DG of the hippocampus. **f** Ketamine administration reversed LPS-induced increase in GluN2B immunoreactivity in CA1 and DG of the hippocampus. **g** Pearson’s correlation coefficient of PSD95 and p-GluN2B was not significantly different among the four groups in CA1, CA3, and DG of the hippocampus. **h** Ketamine administration reversed LPS-induced increase in p-GluN2B immunoreactivity in CA1 and DG of the hippocampus. Scale bars indicate 20 μm. Data are presented as mean ± SEM (*n* = 4–6 mice per group). *p < 0.05, **p < 0.01 vs the Sal + Sal group; ^#^*p* < 0.05, ^##^*p* < 0.01 vs the LPS + Sal group

In the extrasynaptic fractions, ketamine administration reversed LPS-induced increase in the level of GluN2B (Fig. 4a; interaction: LPS × ketamine, *F*_1,12_ = 3.267, *P* > 0.05; LPS: *F*_1,12_ = 7.045, *P* < 0.05; ketamine: *F*_1,12_ = 5.224, *P* < 0.05). In the synaptic fractions, no significant difference in GluN2B level was observed in the four groups (Fig. 4a; interaction: LPS × ketamine, *F*_1,12_ = 0.172, *P* > 0.05; LPS: *F*_1,12_ = 0.020, *P* > 0.05; ketamine: *F*_1,12_ = 0.775, *P* > 0.05). Next, to confirm this finding, dual antibody labeling of surface GluN2B (an antibody that specifically binds to the N-terminal of the GluN2B) and synapse-specific protein PSD95 was used to identify synaptic GluN2B localization. In the CA1, CA3, and DG of the hippocampus, no significant difference in GluN2B/PSD95 colocalization was observed in the four groups (Fig. 4e; CA1: interaction: LPS × ketamine, *F*_1,20_ = 0.109, *P* > 0.05; LPS: *F*_1,20_ = 0.981, *P* > 0.05; ketamine: *F*_1,20_ = 0.303, *P* > 0.05; CA3: interaction: LPS × ketamine, *F*_1,20_ = 2.381, *P* > 0.05; LPS: *F*_1,20_ = 0.000, *P* > 0.05; ketamine: *F*_1,20_ = 0.381, *P* > 0.05; DG: interaction: LPS × ketamine, *F*_1,20_ = 0.000, *P* > 0.05; LPS: *F*_1,20_ = 0.155, *P* > 0.05; ketamine: *F*_1,20_ = 0.843, *P* > 0.05). This result suggests that ketamine did not affect synaptic GluN2B localization. In the CA1 and DG of the hippocampus, ketamine administration reversed LPS-induced increase in GluN2B immunoreactivity (Fig. 4f; CA1: interaction: LPS × ketamine, *F*_1,12_ = 17.929, *P* < 0.05; LPS: *F*_1,12_ = 16.714, *P* < 0.01; ketamine: *F*_1,12_ = 14.044, *P* < 0.01; DG: interaction: LPS × ketamine, *F*_1,12_ = 33.842, *P* < 0.05; LPS: *F*_1,12_ = 52.299, *P* < 0.01; ketamine: *F*_1,20_ = 30.386, *P* < 0.01). Combined with the above results and given that ketamine administration did not affect synaptic GluN2B localization, this result suggests that the ketamine-mediated reduction in GluN2B mainly affects extrasynaptic fractions. In the extrasynaptic fractions, ketamine administration reversed LPS-induced increase in the level of p-GluN2B (Fig. 4b; LPS: *H* = 8.824, *P* < 0.05; ketamine: *H* = 8.824, *P* < 0.05). In the synaptic fractions, no significant difference in p-GluN2B level was observed in the four groups (Fig. 4b; interaction: LPS × ketamine, *F*_1,12_ = 0. 144, *P* > 0.05; LPS: *F*_1,12_ = 0.700, *P* > 0.05; ketamine: *F*_1,12_ = 0.096, *P* > 0.05). In the CA1, CA3, and DG of the hippocampus, no significant difference in p-GluN2B/PSD95 colocalization was observed in the four groups (Fig. 4g; CA1: interaction: LPS × ketamine, *F*_1,20_ = 0.773, *P* > 0.05; LPS: *F*_1,20_ = 0.057, *P* > 0.05; ketamine: *F*_1,20_ = 0.313, *P* > 0.05; CA3: interaction: LPS × ketamine, *F*_1,20_ = 2.381, *P* > 0.05; LPS: *F*_1,20_ = 0.000, *P* > 0.05; ketamine: *F*_1,20_ = 0.381, *P* > 0.05; DG: interaction: LPS × ketamine, *F*_1,20_ = 0.356, *P* > 0.05; LPS: *F*_1,20_ = 0.181, *P* > 0.05; ketamine: *F*_1,20_ = 0.007, *P* > 0.05). In the CA1 and DG of the hippocampus, ketamine administration reversed LPS-induced increase in p-GluN2B immunoreactivity (Fig. 4 h; CA1: interaction: LPS × ketamine, *F*_1,12_ = 21.120, *P* < 0.05; LPS: *F*_1,12_ = 12.588, *P* < 0.01; ketamine: *F*_1,12_ = 11.595, *P* < 0.01; DG: interaction: LPS × ketamine, *F*_1,12_ = 15.625, *P* < 0.05; LPS: *F*_1,12_ = 52.299, *P* < 0.01; ketamine: *F*_1,20_ = 10.632, *P* < 0.01). This result also suggests that the ketamine-induced reduction in p-GluN2B mainly affects extrasynaptic fractions. In summary, these results indicate that downregulation of GluN2B and p-GluN2B induced by ketamine is mainly derived from the extrasynaptic fractions of the hippocampus.

### Ketamine reversed LPS-induced enhancement of the extrasynaptic interaction of p-CaMKIIα–GluN2B in the hippocampus

**Fig. 5:**
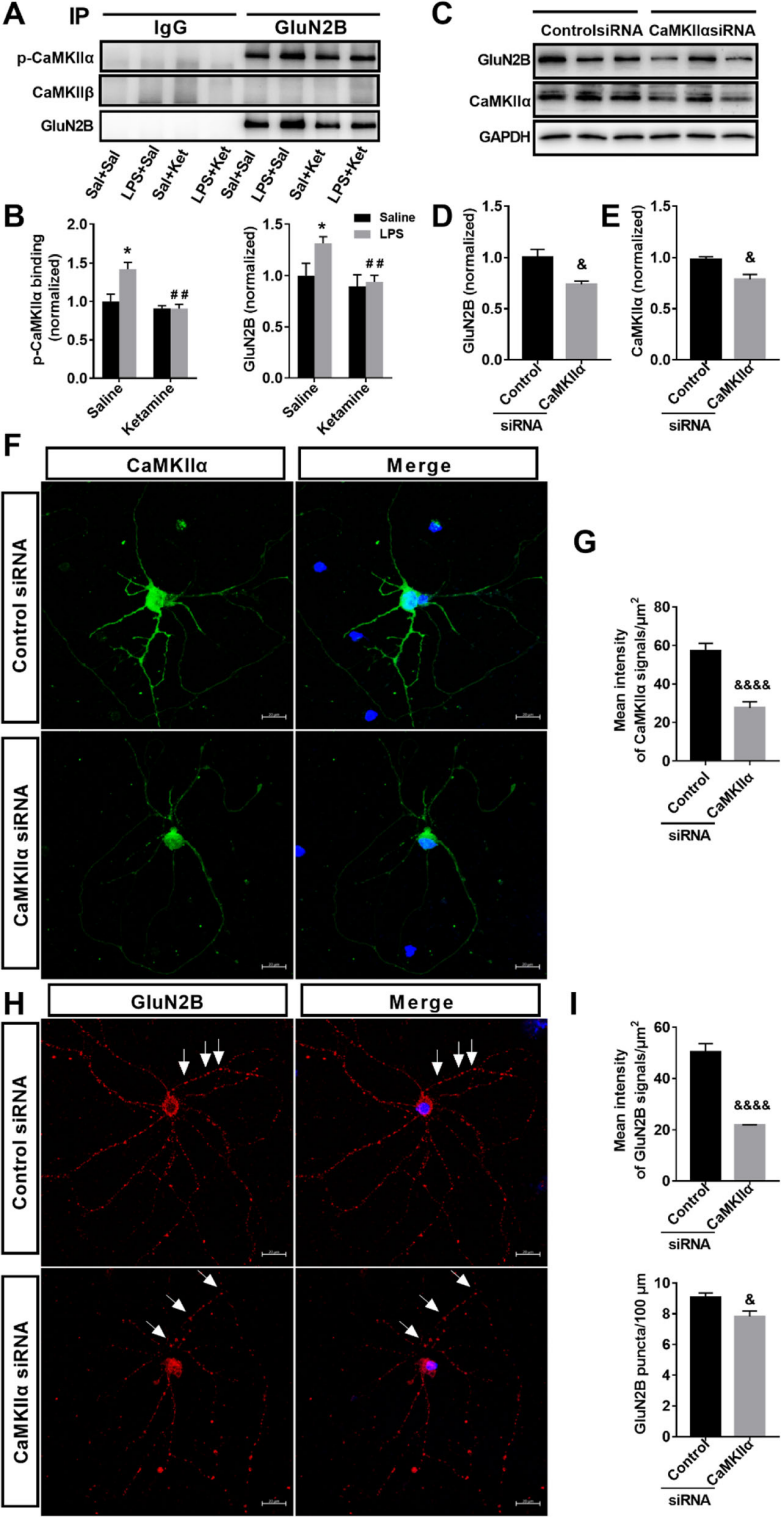
Ketamine reversed the enhanced extrasynaptic p-CaMKIIα–GluN2B interaction in the hippocampus induced by LPS. **a** Immunoprecipitation of GluN2B with p-CaMKIIα and CaMKIIβ in extrasynaptic fractions of the hippocampus. **b** Ketamine administration attenuated the enhanced interaction between p-CaMKIIα and GluN2B induced by LPS. **c** The levels of CaMKIIα and GluN2B in the DIV11 hippocampal neurons after transfection with control siRNA and CaMKIIα siRNA were determined by western blotting. **d** CaMKIIα siRNA decreased GluN2B level in the hippocampal neurons. **e** CaMKIIα siRNA decreased CaMKIIα level in the hippocampal neurons. **f** DIV10 hippocampal neurons were transfected with control siRNA and CaMKIIα siRNA, fixed on DIV11, and immunostained with antibodies to CaMKIIα. Shown are representative confocal images of transfected neurons. **g** CaMKIIα siRNA decreased the mean intensity of CaMKIIα signals in the cell body and dendrites in **f**. **h** DIV10 hippocampal neurons were transfected with control siRNA and CaMKIIα siRNA, fixed on DIV11, and immunostained with antibodies to GluN2B. Shown are representative confocal images of transfected neurons. **i** CaMKIIα siRNA decreased the mean intensity of GluN2B signals in the cell body and dendrite and the GluN2B puncta per 100 μm dendrite length in (**h**). Scale bars indicate 20 μm. Data are presented as mean ± SEM (*n* = 4–6 mice per group or *n* = 60 neurons). *p < 0.05 vs the Sal + Sal group; ^##^*p* < 0.01 vs the LPS + Sal group; ^&^*p* < 0.05, ^&&&&^*p* < 0.0001 vs the control siRNA group

A GluN2B antibody was used to precipitate the NMDA receptors complex from the extrasynaptic fractions of the hippocampus. Immunoprecipitation assays revealed that p-CaMKIIα binds GluN2B receptors in the extrasynaptic fractions of the hippocampus (Fig. 5a). In addition, ketamine administration attenuated the enhancement of the interaction between p-CaMKIIα (interaction: LPS × ketamine, *F*_1,12_ = 8.372, *P* < 0.05; LPS: *F*_1,12_ = 8.207, *P* < 0.05; ketamine: *F*_1,12_ = 16.954, *P* < 0.01) and GluN2B (interaction: LPS × ketamine, *F*_1,12_ = 3.117, *P* > 0.05; LPS: F1_,12_ = 5.341, *P* < 0.05; ketamine: F1,12 = 9.571, *P* < 0.01) induced by LPS (Fig. 5b). These results showed that CaMKIIα was an important binding partner for GluN2B. These results were confirmed by CaMKIIα loss of function assays using siRNA. In cultured hippocampal neurons, after transfection with control siRNA and CaMKIIα siRNA, the efficiency of knocking down was evaluated by western blotting (Fig. 5e; *t* = 3.571, *P* < 0.05) and immunocytochemistry (Fig. 5g; *t* = 5.831, *P* < 0.0001). CaMKIIα siRNA reduced enzyme levels by about 30% (Fig. 5e, g). CaMKIIα siRNA decreased the level (Fig. 5d; *t* = 3.112, *P* < 0.05) and the number and fluorescence intensity of GluN2B puncta (Fig. 5i; *U* = 16, *P* < 0.0001; *t* = 2.516, *P* < 0.05) in the cultured hippocampal neurons. Taken together, these data suggest that the level and number of clusters of GluN2B were regulated by CaMKIIα signal.

### Inhibition of CaMKIIα by KN93 prevented depression-like behaviors and reduced elevated extrasynaptic GluN2B localization and phosphorylation in the hippocampus induced by LPS

**Fig. 6:**
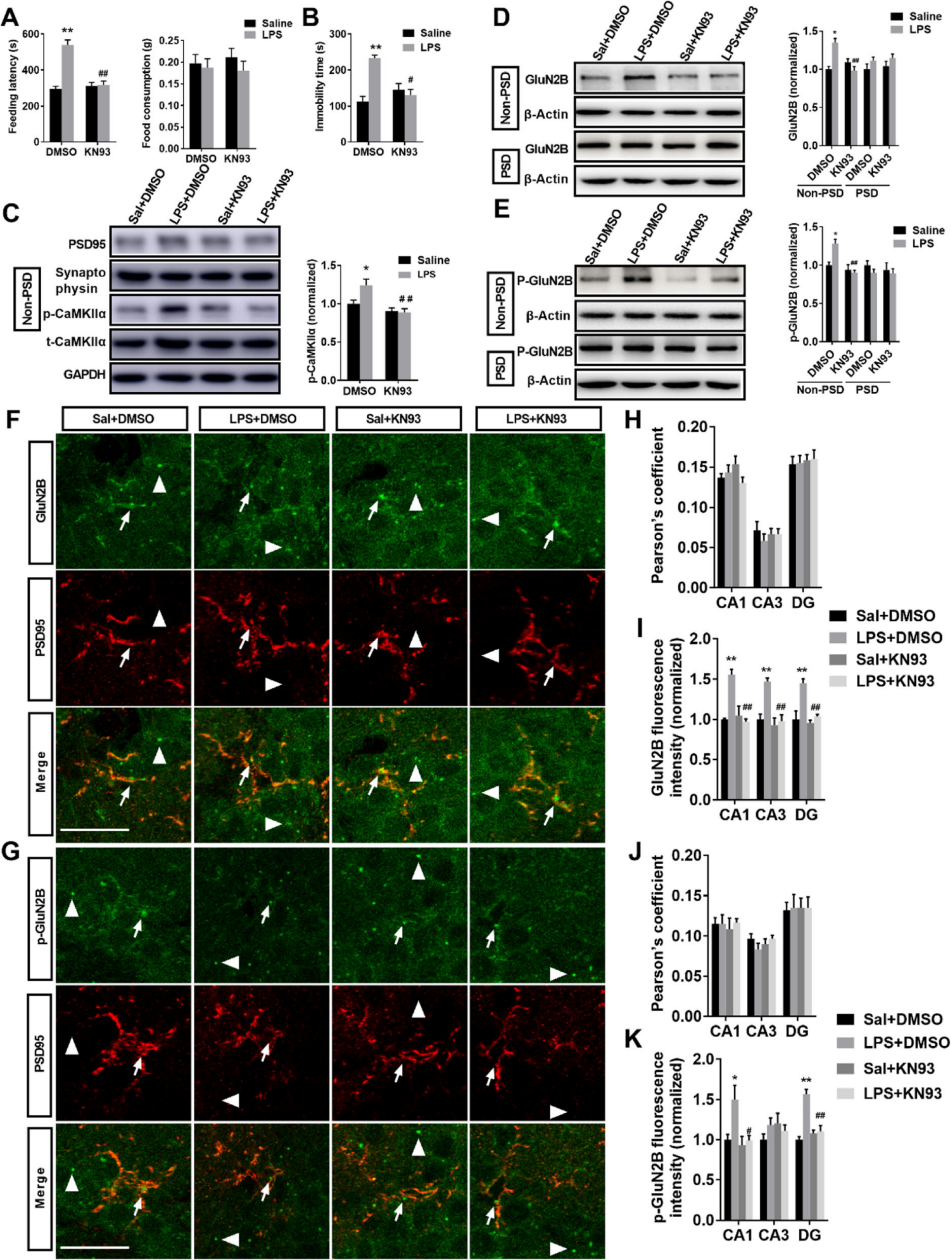
Inhibition of CaMKIIα by KN93 prevented the depression-like behaviors and reduced the elevated extrasynaptic GluN2B localization and phosphorylation in the hippocampus induced by LPS. **a** KN93 treatment prevented LPS-induced increase in feeding latency in the NSFT, and the total food consumption was not different among the four groups. **b** KN93 treatment prevented LPS-induced increase in immobility time in the FST. **c** KN93 treatment reversed LPS-induced increase in extrasynaptic p-CaMKIIα level in the hippocampus. **d** KN93 treatment reversed LPS-induced increase in extrasynaptic GluN2B level but did not affect synaptic GluN2B level in the hippocampus. **e** KN93 treatment reversed LPS-induced increase in extrasynaptic p-GluN2B level but did not affect synaptic p-GluN2B level in the hippocampus. **f** Immunofluorescent images show the colocalization of PSD95 (red) and surface GluN2B receptor (green) in CA1 regions. Arrow indicates colocalization, and triangle indicates no colocalization. **g** Immunofluorescent images show the colocalization of PSD95 (red) with p-GluN2B (green) in CA1 regions. Arrow indicates colocalization, and triangle indicates no colocalization. **h** Pearson’s correlation coefficient of PSD95 and GluN2B was not significantly different among the four groups in CA1, CA3, and DG of the hippocampus. **i** KN93 treatment reversed LPS-induced increase in GluN2B immunoreactivity in CA1, CA3, and DG of the hippocampus. **j** Pearson’s correlation coefficient of PSD95 and p-GluN2B was not significantly different among the four groups in CA1, CA3, and DG of the hippocampus. **k** KN93 treatment reversed LPS-induced increase p-GluN2B immunoreactivity in CA1 and DG of the hippocampus. Scale bars indicate 20 μm. Data are presented as mean ± SEM (*n* = 4–10 mice per group). *p < 0.05, **p < 0.01 vs the Sal + DMSO group; ^#^*p* < 0.05, ^##^*p* < 0.01vs the LPS + DMSO group

To further determine the relationship between the activation of CaMKIIα and GluN2B in vivo, an inhibitor of CaMKIIα, KN93, was used in the present study. In the NSFT, KN93 treatment prevented LPS-induced increase in feeding latency (interaction: LPS × KN93, *F*_1,24_ = 30.107, *P* < 0.01; LPS: *F*_1,24_ = 33.146, *P* < 0.01; KN93: *F*_1,24_ = 21.992, *P* < 0.01; LPS is LPS + DMSO versus Sal + DMSO; KN93 is LPS + KN93 versus LPS + DMSO), and the total food consumption in the four groups were not affected (Fig. 6a; interaction: LPS × KN93, *F*_1,24_ = 0.255, *P* > 0.05; LPS: *F*_1,24_ = 0.954, *P* > 0.05; KN93: *F*_1,24_ = 0.085, *P* > 0.05). Moreover, KN93 treatment prevented LPS-induced increase in immobility time in the FST (Fig. 6b; interaction: LPS × KN93, *F*_1,24_ = 22.909, *P* < 0.01; LPS: *F*_1,24_ = 13.584, *P* < 0.01; KN93: *F*_1,24_ = 5.873, *P* < 0.05). These behavioral results suggest that inhibition of CaMKIIα by KN93 results in an antidepressant phenotype. Furthermore, in the extrasynaptic fractions of the hippocampus, KN93 treatment prevented the increase in p-CaMKIIα levels induced by LPS (Fig. 6c; interaction: LPS × KN93, *F*_1,22_ = 5.312, *P* < 0.05; LPS: *F*_1,22_ = 4.039, *P* < 0.05; KN93: *F*_1,22_ = 15.795, *P* < 0.01). In the extrasynaptic fractions of the hippocampus, KN93 treatment prevented the increase in GluN2B level induced by LPS (Fig. 6d; interaction: LPS × KN93, *F*_1,25_ = 22.571, *P* < 0.05; LPS: *F*_1,25_ = 6.001, *P* < 0.05; KN93: *F*_1,25_ = 8.058, *P* < 0.01). In the synaptic fractions, no significant difference in GluN2B level was observed in the four groups (Fig. 6d; interaction: LPS × KN93, *F*_1,12_ = 0.002, *P* > 0.05; LPS: *F*_1,12_ = 3.494, *P* > 0.05; KN93: *F*_1,12_ = 0.428, *P* > 0.05). In the CA1, CA3, and DG of the hippocampus, no significant difference in GluN2B/PSD95 colocalization was observed in the four groups (Fig. 6 h; CA1: interaction: LPS × KN93, *F*_1,20_ = 3.375, *P* > 0.05; LPS: *F*_1,20_ = 1.042, *P* > 0.05; KN93: *F*_1,20_ = 0.042, *P* > 0.05; CA3: interaction: LPS × KN93, *F*_1,20_ = 0.632, *P* > 0.05; LPS: *F*_1,20_ = 0.632, *P* > 0.05; KN93: *F*_1,20_ = 0.040, *P* > 0.05; DG: interaction: LPS × KN93, *F*_1,20_ = 0.000, *P* > 0.05; LPS: *F*_1,20_ = 0.032, *P* > 0.05; KN93: *F*_1,20_ = 0.289, *P* > 0.05). In the CA1, CA3, and DG of the hippocampus, KN93 treatment reversed LPS-induced increased in GluN2B immunoreactivity (Fig. 6i; CA1: interaction: LPS × KN93, *F*_1,12_ = 21.889, *P* < 0.05; LPS: *F*_1,12_ = 12.144, *P* < 0.01; KN93: *F*_1,20_ = 15.884, *P* < 0.01; CA3: interaction: LPS × KN93, *F*_1,12_ = 8.833, *P* < 0.05; LPS: *F*_1,12_ = 13.404, *P* < 0.01; KN93: *F*_1,20_ = 16.046, *P* < 0.01; DG: interaction: LPS × KN93, *F*_1,12_ = 8.870, *P* < 0.05; LPS: *F*_1,12_ = 18.344, *P* < 0.01; KN93: *F*_1,20_ = 13.806, *P* < 0.01). In the extrasynaptic fractions, KN93 treatment reversed LPS-induced increased in p-GluN2B level (Fig. 6e; interaction: LPS × KN93, *F*_1.12_ = 9.784, *P* < 0.05; LPS: *F*_1,12_ = 5.871, *P* < 0.05; KN93: *F*_1,12_ = 18.922, *P* < 0.01). In the synaptic fractions, no significant difference in p-GluN2B level was observed in the four groups (Fig. 6e; interaction: LPS × KN93, *F*_1,12_ = 0.194, *P* > 0.05; LPS: *F*_1,12_ = 1.157, *P* > 0.05; KN93: *F*_1,12_ = 0.258, *P* > 0.05). In the CA1, CA3, and DG of the hippocampus, no significant difference in p-GluN2B/PSD95 colocalization was observed in the four groups (Fig. 6j; CA1: interaction: LPS × KN93, *F*_1,20_ = 0.169, *P* > 0.05; LPS: *F*_1,20_ = 0.169, *P* > 0.05; KN93: *F*_1,20_ = 0.061, *P* > 0.05; CA3: interaction: LPS × KN93, *F*_1,20_ = 2.609, *P* > 0.05; LPS: *F*_1,20_ = 0.290, *P* > 0.05; KN93: *F*_1,20_ = 0.290, *P* > 0.05; DG: interaction: LPS × KN93, *F*_1,20_ = 0.016, *P* > 0.05; LPS: *F*_1,20_ = 0.16, *P* > 0.05; KN93: *F*_1,20_ = 0.016, *P* > 0.05). In the CA1 and DG of the hippocampus, KN93 treatment reversed LPS-induced increased in p-GluN2B immunoreactivity (Fig. 6 k; CA1: interaction: LPS × KN93, *F*_1,12_ = 3.688, *P* < 0.05; LPS: *F*_1,12_ = 5.904, *P* < 0.05; KN93: *F*_1,20_ = 6.328, *P* < 0.05; DG: interaction: LPS × KN93, *F*_1,12_ = 24.451, *P* < 0.05; LPS: *F*_1,12_ = 29.510, *P* < 0.01; KN93: *F*_1,20_ = 12.616, *P* < 0.01). In summary, these results indicate that CaMKIIα activation has an effect on the localization and phosphorylation of GluN2B in the extrasynaptic fraction of the hippocampus and is related to the rapid antidepressant effect of ketamine.

### Ketamine upregulated the expressions of p-CREB and BDNF and improved the synaptic dysfunction in the hippocampus

**Fig. 7:**
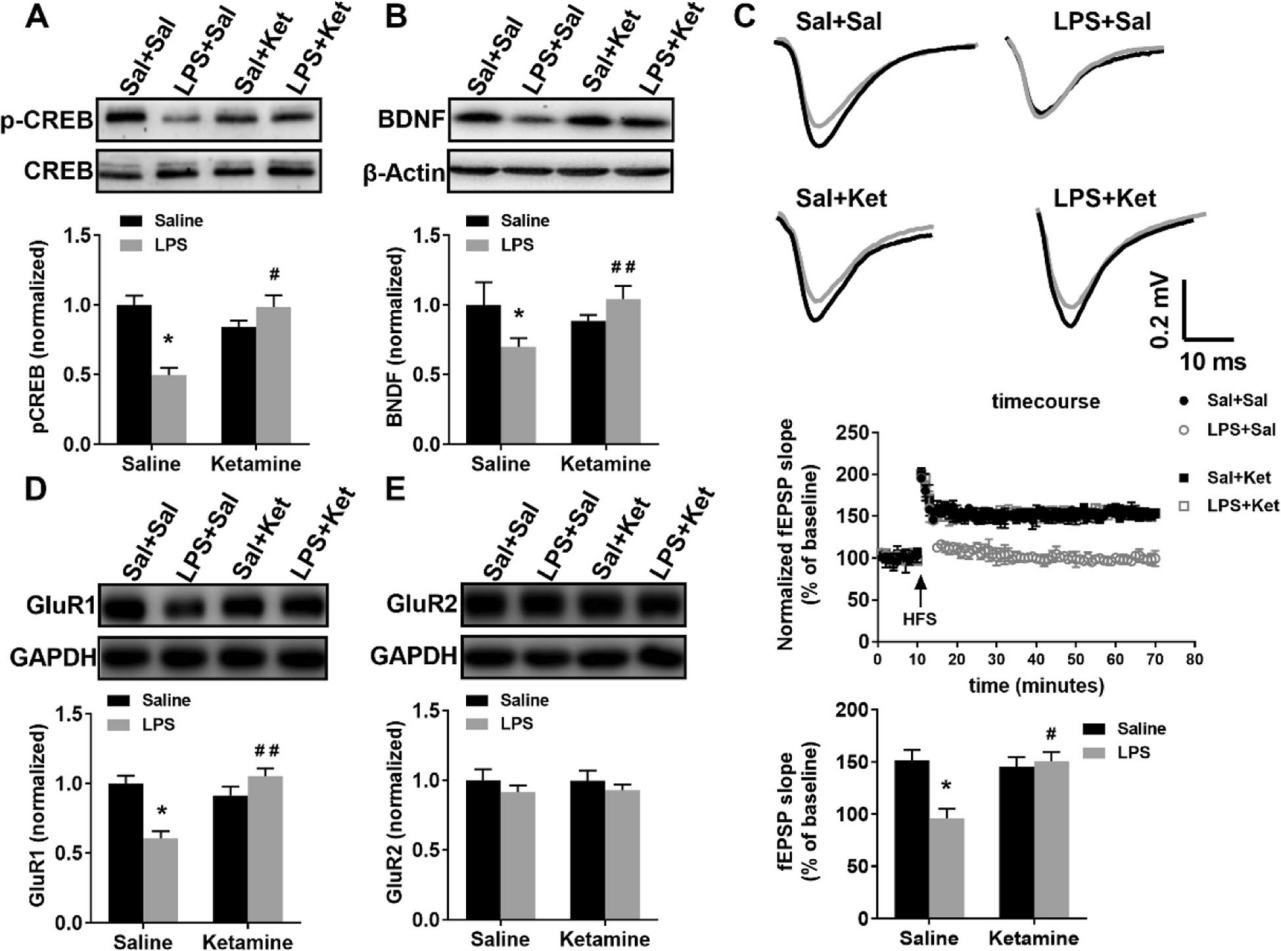
Ketamine reversed LPS-induced downregulation of p-CREB and BDNF expressions and the synaptic dysfunction in the hippocampus. **a** Ketamine administration blocked LPS-induced decrease in p-CREB expression in the hippocampus. **b** Ketamine administration blocked LPS-induced decrease in BDNF expression in the hippocampus. **c** Schematic representation of fEPSP before (gray) and after (black) HFS among the four groups. Horizontal calibration bars, 10 ms; vertical bars, 0.2 mV. Time course of fEPSP data normalized to the level of the 10-min baseline interval. Ketamine administration prevented LPS-induced depression of SC-CA1 LTP in the hippocampus. **d, e** Ketamine administration reversed LPS-induced decrease in GluR1 level but did not affect GluR2 level in the hippocampal synaptosomes. Data are presented as mean ± SEM (*n* = 4-7 mice per group). *p < 0.05 vs the Sal + Sal group; ^#^*p* < 0.05, ^##^*p* < 0.01 vs the LPS + Sal group

Ketamine administration blocked LPS-induced significantly decreased in p-CREB expression in the hippocampus (Fig. 7a; interaction: LPS × ketamine, *F*_1,12_ = 25.310, *P* < 0.05; LPS: *F*_1,12_ = 7.936, *P* < 0.05; ketamine: *F*_1,12_ = 6.672, *P* < 0.05). Ketamine administration blocked LPS-induced significantly decreased in BDNF expression in the hippocampus (Fig. 7b; interaction: LPS × ketamine, *F*_1,12_ = 22.039, *P* < 0.05; LPS: *F*_1,12_ = 6.525, *P* < 0.05; ketamine: *F*_1,12_ = 16.408, *P* < 0.01). Ketamine administration sufficiently prevented LPS-induced depression in SC-CA1 LTP of the hippocampus (interaction: LPS × ketamine, *F*_1,8_ = 10.706, *P* < 0.05; LPS: *F*_1,8_ = 7.476, *P* < 0.05; ketamine: *F*_1,8_ = 6.972, *P* < 0.05). Ketamine administration reversed the decrease in GluR1 level induced by LPS but did not affect GluR2 level (Fig. 7d, e; GluR1: interaction: LPS × ketamine, *F*_1,12_ = 21.165, *P* < 0.05; LPS: *F*_1,12_ = 4.478, *P* < 0.05; ketamine: *F*_1,12_ = 9.843, *P* < 0.01; GluR2: interaction: LPS × ketamine, *F*_1,12_ = 0.019, *P* > 0.05; LPS: *F*_1,12_ = 1.425, *P* > 0.05; ketamine: *F*_1,12_ = 0.010, *P* > 0.05).

## Discussion

**Fig. 8:**
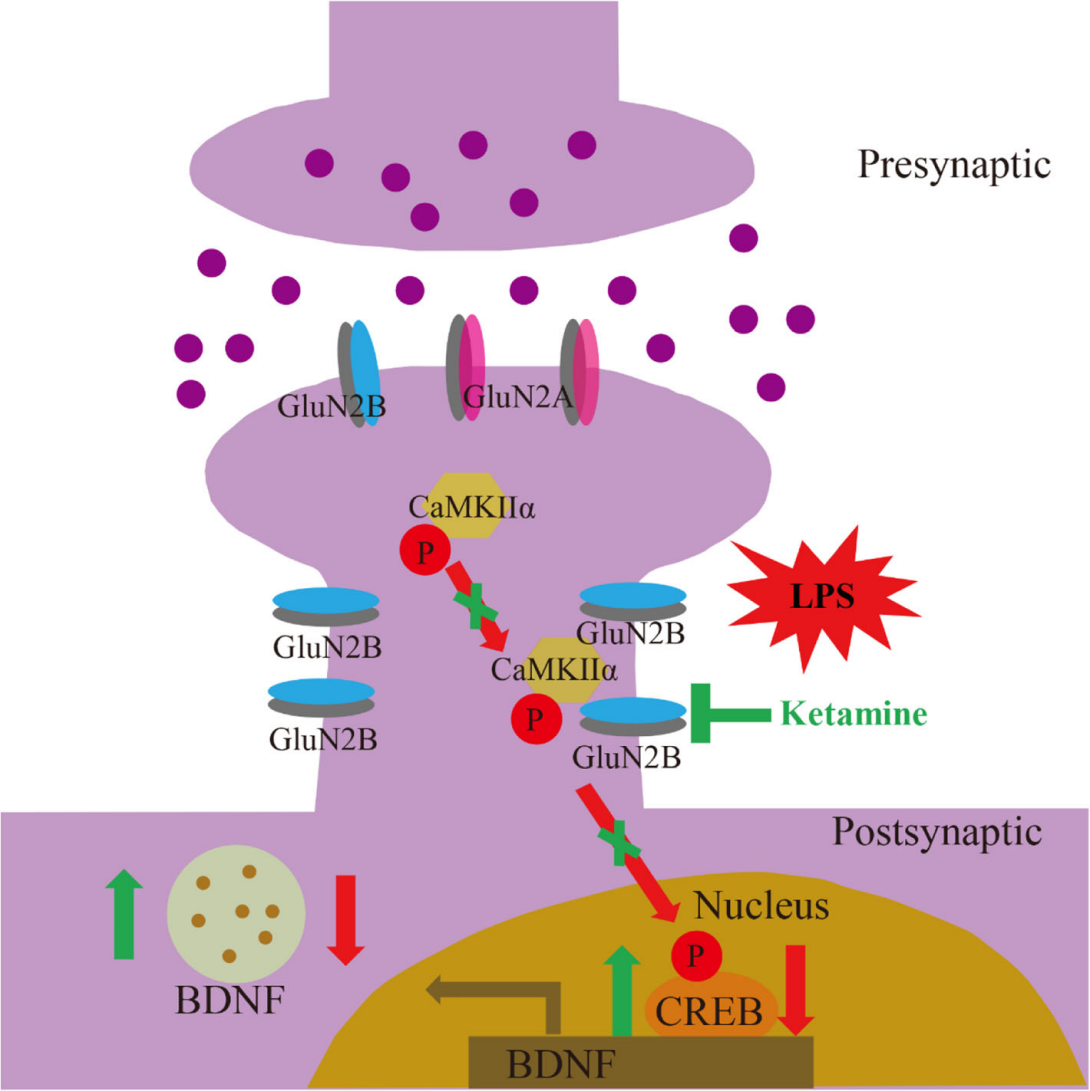
Schematic diagram illustrates extrasynaptic CaMKIIα and GluN2B in the hippocampus are involved in the antidepressant process of ketamine. LPS stimulation induces extrasynaptic CaMKIIα phosphorylation (p-CaMKIIα) and then binding to extrasynaptic GluN2B in the hippocampus, which causes extrasynaptic GluN2B retention and phosphorylation. The activation of extrasynaptic GluN2B downregulates p-CREB and BDNF levels and subsequently impairs LTP induction as well as induces synaptic protein deficits in the hippocampus, ultimately leading to depression-like behaviors. Ketamine administration reverses extrasynaptic CaMKIIα activation, followed by the normalization of extrasynaptic GluN2B localization and phosphorylation, which increases p-CREB, BDNF, and GluR1 levels in the hippocampus, and finally rescues depression-like behaviors

In the present study, we found decreased extrasynaptic CaMKIIα activity and the resulting changes in extrasynaptic GluN2B localization and phosphorylation were associated with the antidepressant effects of ketamine. Furthermore, ketamine administration upregulated the expressions of p-CREB and BDNF in the hippocampus and prevented the impairment of LTP induction as well as the synaptic protein loss induced by LPS. Altogether, these results suggested that extrasynaptic CaMKIIα plays a key role in ketamine’s antidepressant effects by affecting extrasynaptic GluN2B localization and phosphorylation, and synaptic plasticity. Thus, we propose a model describing a possible pathway for extrasynaptic CaMKIIα to participate in the antidepressant mechanism of ketamine (see Fig. 8).

The prevalence of depression is higher in patients with immune disorders and infections, suggesting inflammation plays a key role in depression. For this reason, we established an animal model of depression by LPS injection, which has been well described in previous studies [26]. It has been reported that LPS at a dose of 0.83 mg/kg could successfully induce depression-like behaviors [27]. In our study, depression-like behaviors were induced by LPS (1 mg/kg) at 24 h after intraperitoneal injection, which were prevented by ketamine administration. Furthermore, LPS injection induced anxiety behaviors, which were also reversed by ketamine administration. Moreover, ketamine administration improved the deteriorating state of fur coat of mice induced by LPS. The latter is also a well-validated index of depression [28]. Similarly, a previous study reported that antidepressant CRF1 antagonist could counteract the deleterious effects of chronic unpredictable stress on coat state [29]. Taken together, our results further confirm prior observations of antidepressant and anti-anxiety effects of ketamine [30].

Neuroinflammation is considered as a risk factor for depression and also a marker of resistance to depression treatment [31, 32]. Increased levels of proinflammatory biomarkers (including CRP, IL-6, and TNF-α) were found in patients with depression [33, 34], whereas NSAIDs treatment significantly inhibited the inflammatory response and reduced the suicidal ideation and depression symptoms in depressed patients [35]. Similarly, ketamine administration has been shown to suppress the LPS-induced inflammatory response in human [36] or rats [37] and reduce suicidal symptoms and HDRS scores in depressed patients [38]. Furthermore, previous studies have shown that ketamine could alleviate LPS-induced depression-like behaviors, which were related to increased expressions of IL-1β and IL-6 and decreased expression of IL-10 in the rats [39]. Moreover, serum IL-6 is considered a predictive biomarker for ketamine’s antidepressant effect in treatment-resistant patients with depression [40]. In animal studies, it has been demonstrated that there is a close relation between microglia and synaptic plasticity. Microglia dynamically interact with synapses and participate in activity-dependent synaptic pruning and maturation [41]. In human studies, significant microgliosis is observed in depressed patients with suicide [42]. Classical antidepressants could inhibit microglial responses caused by inflammatory stimulus [43]. However, the mechanism by which inflammation induces depression remains unclear.

Accumulating evidence suggests that CaMKIIα is involved in the therapy for depression. Chronic fluoxetine treatment reduces the activation of CaMKIIα neurons in the mPFC, reversing the effect of poststroke depression [44]. The inhibition of CaMKIIα neurons in the lateral habenula underlies the antidepressant effects of light therapy [45]. Furthermore, intraperitoneal injection of TATCN21, an inhibitor of CaMKIIα, shows antidepressant phenotype in two classic depression-related behavioral tests [8]. Similarly, in the present study, ketamine administration reversed extrasynaptic CaMKIIα activation in the hippocampus. Moreover, a CaMKIIα inhibitor, KN93, also reversed extrasynaptic CaMKIIα activation and attenuated depression-like behaviors. Currently, no previous studies investigated the role of extrasynaptic CaMKIIα in LPS-induced depression-like behaviors and the antidepressant effect of ketamine. Most studies focus on the total level of CaMKIIα in different brain regions. Our results showed that changes in CaMKIIα activation were observed in extrasynaptic but not in the synaptic fraction. A recent study showed that xanthoceraside alleviated learning-memory deficits in APP/PS1 transgenic mice, and the mechanism is associated with increased expression of CaMKII in synaptic, but not extrasynaptic fraction [46]. In addition, previous studies have shown that the anxious-like behaviors in serotonin receptor 1a knockout mice are related to the increased phosphorylated CaMKIIα in extrasynaptic fraction of the hippocampus [47]. Although the function of extrasynaptic CaMKIIα is not fully understood, we speculated that increased phosphorylated CaMKIIα in extrasynaptic fraction of the hippocampus might contribute to LPS-induced depression-like behaviors.

The activation of extrasynaptic NMDARs has an important role in the etiology of depression [48]. Chronic unpredictable stress caused overactivation of extrasynaptic GluN2B in the mPFC and the GluN2B antagonist produced rapid antidepressant-like effects [24]. Moreover, blocking of extrasynaptic NMDA receptors is also involved in the antidepressant effects of ketamine by preventing ElF-2 phosphorylation and BDNF synthesis [49]. Similarly, in our study, ketamine administration reduced extrasynaptic GluN2B localization and phosphorylation in the hippocampus. Changes in GluN2B are observed in extrasynaptic, but not in the synaptic fraction. Indeed, this selectivity has been noted and described in a review of the mechanisms of ketamine action as an antidepressant [48]. In addition, another NMDAR antagonist, memantine, preferentially blocked the extrasynaptic NMDAR currents rather than synaptic currents [50]. In general, CaMKIIα is involved in physiological processes by interacting with various substrate molecules. It has been reported that CaMKIIα could stay at the postsynaptic site for a longer time by binding to GluN2B, and this interaction was considered to be crucial for synaptic structure and strength [51, 52]. The interaction between CaMKIIα and GluN2B in the extrasynaptic fraction of the hippocampus was not well understood, while the location of the protein was different, and its role in participating in physiological and pathological processes was also different [53, 54]. In the present study, ketamine administration attenuated the enhanced interaction between p-CaMKIIα and GluN2B in the extrasynaptic fraction of the hippocampus. CaMKIIα siRNA reduced the level and number of puncta of GluN2B in cultured hippocampal neurons. In addition, inhibition of CaMKIIα activity by KN93 reduced extrasynaptic GluN2B localization and phosphorylation in the hippocampus. Similarly, it has been shown that in a model of LPS-induced neuronal injury, GluN2B antagonists could reverse neuronal damage by disrupting a signaling complex such as GluN2B–CaMKII–PSD95 and further reducing the level of phosphorylated CaMKII [55]. In general, Ca^2+^ can flow into the cell through the GluN2B-containing NMDA receptors on the membrane, causing phosphorylation of CaMKIIα at the Thr286 site. Subsequently, activated CaMKIIα can phosphorylate the GluN2B receptor, further promoting Ca^2+^ influx into the cell. Therefore, CaMKIIα and the GluN2B receptor may interact in a positive feedback manner. Our data raise a possibility that CaMKIIα/GluN2B in the extrasynaptic fraction might play a key role in the antidepressant effects of ketamine by accelerating Ca^2+^ flow into the cell and causing neuronal dysfunction. Previous studies have showed that GluN2B-containing NMDA receptors are essential for ketamine’s antidepressant effects and GluN2B antagonist could rapidly reverse depressive-like behavior [11], our results may complement previous findings that extrasynaptic CaMKIIα is also involved in ketamine’s antidepressant effect, with downstream involvement of GluN2B.

Normal NMDARs activity is crucial to neurotransmission [56]. Excessively enhanced NMDARs activity can lead to intracellular calcium overload and trigger a series of pathological processes, including depression [57]. The specific effects of NMDARs activation depend on the subcellular localization of the receptor [58]. Synaptic NMDARs activation promotes CREB phosphorylation, whereas extrasynaptic NMDARs activation decreases CREB phosphorylation [59]. Furthermore, the BDNF gene is controlled by several transcription factors, and CREB plays a central role in this regulatory process [60]. Antidepressants can increase BDNF levels in the hippocampus, while viral knockout of BNDF in specific subregions of the hippocampus can induce depression-like behaviors [61]. In the present study, LPS injection decreased the levels of p-CREB and BDNF in the hippocampus, which were reversed by ketamine administration. These results suggested that CREB inhibition pathway induced by overactivated extrasynaptic GluN2B participated in ketamine’s antidepressant effects.

The disruption of synaptic plasticity is considered to be one of the neurobiological manifestations of depression [62]. AMPA receptors play a key role in the formation, maintenance, and homeostasis of synaptic plasticity [63]. It has been shown that AMPARs can be quickly transported to synapses during LTP [64]. Adult knockout mice lacking GluR1 do not show LTP, suggesting that the GluR1 subunit is essential for the production of LTP. It has been reported that fluoxetine can enhance the LTP in the hippocampus [65]. AMPAR subunits (GluR1 and GluR2) are upregulated in the hippocampus after ketamine administration [19]. Pre-treatment with the AMPAR antagonist NBQX can block the antidepressant effects of ketamine in animal models of depression [66, 67]. In the present study, ketamine prevented the impaired LTP induction induced by LPS. Moreover, it also significantly increased the level of GluR1 but did not alter the level of GluR2 in the hippocampal synaptosomes. These results suggested that ketamine’s antidepressant effects shifts from early functional plasticity to late structural homeostasis [48].

There are some limitations in our study. Firstly, we did not observe long-term changes in CaMKIIα after ketamine administration, which needs to be confirmed in further study. Secondly, clinical and animal studies have shown that the antidepressant effects of ketamine are gender dependent [68, 69]. Moreover, women are twice as likely as men to suffer from major depression [70]. Therefore, further study of ketamine is also required on gender differences. Thirdly, we did not verify whether overexpression of CaMKIIα could block ketamine’s antidepressant effects, which needs to be confirmed in our future study. Finally, it should be noted that the number of animals in our study is relatively small, we will include more mice in our further studies to make the evidence more reliable.

## Conclusion

In conclusion, our results indicate that ketamine is effective in reversing LPS-induced depression-like behaviors, a process associated with decreased extrasynaptic CaMKIIα activity in the hippocampus. This further induces disturbance of extrasynaptic GluN2B localization and phosphorylation, which subsequently affected synaptic plasticity and induces depression-like behavior. Taken together, our study provides additional evidence that extrasynaptic CaMKIIα is involved in the antidepressant effects of ketamine by downregulating GluN2B receptors in an LPS-induced depression model, which will contribute to the development of the next generation of more effective and safer antidepressants.

## Data Availability

The data that support the findings of this study are available from the corresponding author upon reasonable request.

## Abbreviations

ACSF: Artificial cerebrospinal fluid
BCA: Bicinchoninic acid
CaMKII: Calcium/calmodulin-dependent protein kinase II
CRP: C-reactive protein
CRF1: Corticotropin-releasing factor 1
DMSO: Dimethyl sulfoxide
fEPSP: Field excitatory postsynaptic potential
FST: Forced-swimming test
HFS: High-frequency stimulation
HDRS: 17-item Hamilton Depression Rating Scale
IL-6: Interleukin 6
IL-10: Interleukin 10
IL-1β: Interleukin 1 beta
KN93: C26H29ClN2O4S.H3O4P
LPS: Lipopolysaccharide
NSAIDs: Nonsteroidal anti-inflammatory drugs
NMDARs: *N*-methyl-d-aspartate receptors
NSFT: Novelty-suppressed feeding test
OFT: Open-field test
PSD95: Postsynaptic density-95
TNF-α: Tumor necrosis factor-alpha
